# As to Proprietary Medicines

**Published:** 1906-06

**Authors:** 


### As to Proprietary Medicines.

The bitter and selfish fight being made by the *Journal of the American Medical Association,* some of its satellites, and a few lay papers, on pharmaceutical preparations, has degenerated to a point, where misrepresentation and positive mendacity have become their chief stock in trade. While an honest and upright fight was being made for what was supposed by some to be a principle of medical ethics, we were willing to let the matter run its course, knowing and believing that right would in the end prevail. But where misrepresentation and other unfair means are used to inflame and mislead the profession and the public, we think it time for us to say something about the matter and place ourselves on record, by setting forth the *truth* regarding these attacks, in so far as it appears to us.

For instance, *Collier's Weekly* published a list of 22 cases of poisoning "from headache remedies" and this was referred to gloatingly by the *Journal of the American Medical Association* and other medical journals. Yet not one of them all had the grace, fairness, or honesty to later publish the fact, that a careful investigation of these 22 cases by the *Western Druggist,* and the *National Druggist* disclosed that only *four* cases were bona fide, the remaining 18 cases of death from morphin, strychnin, arsenic, Bright's disease, and other diseases, not in the remotest way connected with headache remedies. One of these trumped-up cases was even found to have been caused by a gun shot wound.

From time to time, apparently inspired reports of deaths or serious illness from the coal-tar relievers appear in the daily press, and investigations of these reports almost invariably show them to be absolutely without a basis of fact, or to have so little truth behind them as to prove beyond peradventure of a doubt, that they are inspired by some one whose purpose it is to mislead, regarding the safety of these remedies, if properly used.

The very evident purpose of these reports is to create alarm, *regardless of the facts,* touching the coal-tar preparations, so that when the State Legislatures are in session next winter, the *Ladies' Home Journal* bill and other similar bills, classing the coal-tar preparations as poisons, will pass the various legislatures. We are pleased to say that this scheme to create a fear of coal-tar preparations by *false reports,* will fail, because statistics are being carefully compiled, showing the number of such cases *falsely* reported or greatly exaggerated. These statistics, presented to the various

State legislatures where such bills are under consideration, expose the underhanded methods being used, and will act as a decided boomerang to those who consider it honorable to use disreputable means to accomplish their ends.

The coal-tar derivatives appear to be the particular butt of attack by those who are creating the present epidemic of hysteria regarding these remedies and the most ridiculous and previously unheard-of statements are made concerning them. Already bills have been introduced in several State legislatures classing all coal-tar derivatives as poisons, yet *Collier's,* assisted by the *Journal of the American Medical Association* was, as stated above, able to find only four bona fide cases of untoward effect resulting from *overdoses of all the various coal-tar preparations sold in the United States and Canada.* Yet there have been recorded over 100 cases of quinin blindness, numberless cases of quinin deafness, thousands of cases of skin lesions and other untoward results from small doses of quinin, not to say anything of the numerous deaths caused by it. And quinin is considered a *safe* remedy, and is not mentioned in the Schedule of Poisons referred to in the above-mentioned bills.

The coal-tar preparations are not poisons, as that term is usually understood, and every person familiar with medical literature knows—though all may not publicly admit it—that there are more cases of poisoning by almost every potent drug, than by all the various coal-tar preparations combined. This, in spite of the fact that there are probably more doses of the coal-tar pain relievers taken every day, than are taken of any other five drugs.

Some of these preparations we have been using for a number of years and with every degree of satisfaction and most gratifying results. One of which in particular, known as Antikamnia, has been on the market for over fifteen years, and during all that time, not *one* well authenticated case of untoward effect has ever been reported where the proper and advised dose was taken. To call a preparation "Poison" which never acts even unpleasantly, unless taken in greatly excessive doses, is ridiculous, to say the least. Salt in excessive doses is a poison, and has often produced death. All food contains the most powerful poisons in minute quantities; for instance, prussic acid in fruits, other poisonous substances in meat, fish, eggs, milk, bread, beer, many vegetables, mineral waters, etc. Therefore these articles of food and drink could more justly be called "poison" than to call a medicine a poison simply because it contains such drugs or a coal-tar preparation. It is essential that a certain prescribed quantity be exceeded to convert any substance into a poison. However, all drugs which are poisonous when given to human beings in doses of a single grain or less, are commonly called "poisons" and these drugs are the substances that people think of when poison is mentioned. To call other drugs "poisons" is misleading, unjust, and is evidence of pure malice.

Of all dishonorable and unprincipled means to injure an article of trade, be it medicine or other substance, the worst is that which libels its character. Numbers of pounds of the coal-tar preparations have been administered in the past twenty years, with injurious results from overdoses so rare as to be the marvel of medical history: The New York Quinine & Chemical Company alone, sells over 100 tons of acetanilid a year. No other potent drug is as safe as these remedies, and those who are attempting, from ulterior motives, to create the impression that they are poisons, are doing an act for which all right-minded people ought to, and will, condemn them.

"What is a Poison?" is the title of a very able article by Dr. R. G. Eccles, published in *American Medicine* on December 9 and 16, 1905. Every medical man and especially every editor of a medical journal should read the said article, now that the attempt is being made to classify the coal-tar preparations as poisons, and he will see the utter absurdity and the plainly dishonest motive behind the work being done to misrepresent these safe and efficacious remedies. If the coal-tar preparations are poisons, then quinin, mur. ammonia, chlorate of potash, calomel, sodium bicarb., santonin, and the bromides are poisons as well, as are many other drugs which are now considered safe remedies, and which are never referred to as poisons, in the common acceptation of that term.

We have used quite freely *Antikamnia* in our remarks, for the reason of its great popularity and the universally good results that have been obtained by its judicious and proper use. But there are others that have been unjustly decried and the effort has been made to put under the ban anything and everything, no matter what, just so that it is a "Proprietary." Our medical education was obtained at the feet of a Gamaliel in the profession, one of the most ethical of the ethical, Dr. Wm. K. Bowling, aided by personal instruction from his colleagues, Drs. Paul F. Eve and Wm. T. Briggs, all of this city and all of them at one time presidents of the American Medical Association. We were taught by them that it was a duty to use anything under the heavens above, on the earth beneath, or under the latter and in the waters thereon, yes, anything that in our opinion or belief would relieve pain and suffering and stay the hand of death. Regular medicine has no bounds to the domains from which it is justifiable in procuring materials to aid the injured, the sick, and the suffering. It is absolutely unlimited in the fields from which it may obtain remedial measures or substances for the "relief of any of the ills that flesh is heir to."

Briefly and cursorily I will mention other "Proprietaries" concerning which, having had satisfactory information, I was justified in making trial, and found them by personal observation to be thoroughly reliable and satisfactory; and consequently shall continue to use them, notwithstanding they are decried by a few extremists following in the lead of one who is so very extra ethical for the same reason that a thief will often be the first one to raise the cry of "stop, thief" to ward off suspicion from himself. But of this more anon.

Now, there is "Fellow's Hypophosphites," which I use and have used for years, from the fact that it is far easier to write than it is to put down in detail the various ingredients in the old formula of Churchill; and then, again, I am confident that by doing so there is far less liability of getting inefficient ingredients than I would if I had written the formula and sent it to many of even the leading prescription pharmacies in any city. And not only is it of definite strength as to all its component parts, all of which I can depend on as being carefully selected, but it will always cost my patients less. The same may be said if I prefer to write "Robinson's Hypophosphites."

I use Tongaline because I know that it contains the salicylic acid from the oil of wintergreen and never the synthetic preparation. Furthermore, I know from repeated trial that it is a most excellent remedy in neuralgic conditions in which I want the benefit of this acid together with colchicum, etc.

Battle's Bromidia contains in each fluidounce a definite quantity of chemically pure bromides, chloral, and cannabis indica, enabling me to give a suitable and sufficient dose in a teaspoonful, which I have never been able to accomplish in the best retail pharmacies in this or any other city. I have also found Battle's Iodia, Papine, and Ecthol most excellent and thoroughly reliable preparations—just as much so as "McMunn's Elixir," or any of the "officinal preparations" of the U. S. P.

More than twenty years ago, in a consultation with Dr. John H. Callender of this city, then Superintendent of the Hospital for the Insane in the middle division of this State, he suggested, taking a copy of the prescription from his memorandum book written by the late Dr. Jno. P. Gray, then Superintendent of the Utica Asylum of New York, a tonic combination that is now put up under the "copyrighted" name of "Gray's Glycerine Tonic," and which I use very often, finding it more uniform in its definite strength and more reasonable in price than I ever did during the years I used it and had it compounded by the best retail prescription houses in this city.

Hayden's Viburnum Compound, Wayne's Elixir, etc., are but the well tried and definitely proven working prescriptions of able and active regular practitioners of medicine, far better prepared and more reasonable in price than if one should write out and depend on any prescription druggist for compounding the original formula.

Antiphlogistine has relieved my patients and their friends from the many annoyances of the old-time poultice, giving better results. Listerine is so well established as a "definite and standard" combination, as is Glyco-Heroin (Smith), Glyco-Thymoline, Cystogen, and others that they will long withstand the attacks that have been made on "proprietary medicines."

But why this "hue and cry" that has recently been raised as to "copyrighted" or "proprietary" medicines? The enterprising managers of *Collier's Weekly* and the *Ladies' Home Journal,* in order to get the advantage in a purely business point of view of other periodicals of like character, commenced a crusade against patent and proprietary medicines. Regardless as to whether they were good, bad, or indifferent, they made a wholesale attack. They simply struck out on new lines, expecting to find "pay dirt." It was strictly a matter of exploiting in a new field, expecting to realize thereon in a purely financial way. Well, that is their privilege, provided that they do not resort to *libel.*

Now, as to the *Journal of the American Medical Association.* In 1857 we had the honor of being present at a meeting of the American Medical Association held in this city, being then in our novitiate; and in 1876 became a member of the Association at its Louisville meeting, serving as a delegate to the Association at the meeting in 1877 in Chicago, and a member of the Nominating Committee that year and in several subsequent years. For ten years we missed attendance on but few meetings of the Association. A little over a quarter of a century ago, the *Journal of the American Medical Association* was established, and I have in my possession several letters from the "Father of the Association" in his own handwriting acknowledging my humble efforts in this movement. Of late I have not been present at many of the meetings of the Association, but have had the pleasure of "keeping in touch" with it, although for the most part, being but a "looker-on in Venice," yet my interest therein has been continuous and sincere. The *Journal* under the management of its able, efficient, and "ethical" editors, Dr. N. S. Davis, J. C. Culbertson, Jno. Hollister, and Jno. B. Hamilton grew apace and became "a power in the land."

Yet, in the years and under the editorial management of these "regular members of the medical profession," it accepted the advertisements of all the proprietary preparations mentioned previously and some others. These *"redacteurs en chef,"* being "like Cæsar's wife," were not afraid to admit to its advertising space preparations that had proved of benefit to many in the land. But at a later day, one who had received his diploma from a Homeopathic school, who had "claimed a special designation and traded on the same," one who had been practicing sectarian medicine, was placed on the editorial tripod of the then greatest medical journal in America, and at once, although placed in this high and responsible position by the "Board of Trustees," began his control of them. Yes, it was and is a case of "the tail wagging the dog." And, "out-Heroding Herod," under the inspiration of *Collier's* and the *Ladies' Home Journal,* as has before been done by the guilty thief, he raises the cry of "STOP, THIEF!"

Under his management he would strike down reputable manufacturers who had been recognized as honest and reputable by his able predecessors in so responsible a position, and wherefore? Has he become uneasy as to his tenure of office? Is he uneasy lest some one dig the ground from under his feet? Is it for love, affection for, and sincere devotion to "Regular Medicine"? There is a motive in all things that inspire men to action. What is his? He is shrewd, he is sharp, but his antecedents did not justify his being placed in the editorial chair of the official organ of the regular profession. Will he be sustained in the days that are coming?

We know full well that the charge will be made that this editorial article is inspired by the financial consideration of the advertising pages of this journal. To this we will say, that in all the years that have passed since the establishment of the *Journal of the American Association* we have held as our criterion of the proprietary preparations the *Journal* of the Association. If an advertisement appeared in its pages, we could feel that we were doing no wrong in admitting it to ours. And, as before stated, when we know that any article or combination of chemicals and drugs will prove beneficial to the sick and suffering, we will not hesitate to use them, and also to advocate their use by others, notwithstanding what has been said or may be said by some very small per-SIMMONS.—*Editorial in May Number of The Southern Practitioner, Nashville, Tenn.*

